# nuID: a universal naming scheme of oligonucleotides for Illumina, Affymetrix, and other microarrays

**DOI:** 10.1186/1745-6150-2-16

**Published:** 2007-05-31

**Authors:** Pan Du, Warren A Kibbe, Simon M Lin

**Affiliations:** 1 Robert H. Lurie Comprehensive Cancer Center, Northwestern University, Chicago, IL, 60611, USA

## Abstract

**Background:**

Oligonucleotide probes that are sequence identical may have different identifiers between manufacturers and even between different versions of the same company's microarray; and sometimes the same identifier is reused and represents a completely different oligonucleotide, resulting in ambiguity and potentially mis-identification of the genes hybridizing to that probe.

**Results:**

We have devised a unique, non-degenerate encoding scheme that can be used as a universal representation to identify an oligonucleotide across manufacturers. We have named the encoded representation 'nuID', for nucleotide universal identifier. Inspired by the fact that the raw sequence of the oligonucleotide is the true definition of identity for a probe, the encoding algorithm uniquely and non-degenerately transforms the sequence itself into a compact identifier (a lossless compression). In addition, we added a redundancy check (checksum) to validate the integrity of the identifier. These two steps, encoding plus checksum, result in an nuID, which is a unique, non-degenerate, permanent, robust and efficient representation of the probe sequence. For commercial applications that require the sequence identity to be confidential, we have an encryption schema for nuID. We demonstrate the utility of nuIDs for the annotation of Illumina microarrays, and we believe it has universal applicability as a source-independent naming convention for oligomers.

**Reviewers:**

This article was reviewed by Itai Yanai, Rong Chen (nominated by Mark Gerstein), and Gregory Schuler (nominated by David Lipman).

## Background

Oligonucleotides (often simply referred to as oligos or oligomers) are typically between 25 to 75 bases long. Microarray manufacturers have extensively used oligos as sequence-specific probes to detect the expression of genes. An oligo sequence that is located on a specific bead or position on a microarray is usually referred to as the probe sequence, or just as the probe. Current microarrays by Affymetrix, Agilent, Illumina and others have tens of thousands or even hundreds of thousands of unique sequence probes in an array [1].

In comparison with the stable identifiers for genes that are available through GenBank/EMBL/DDBJ, a stable naming convention or identification scheme for probes has not been established. As a result, some manufacturers have internally created oligonucleotide identifiers, while others have reused external gene identifiers for oligos. Neither of these solutions has been ideal.

With proprietary identifiers, oligo probes that are sequence identical may have different identifiers between manufacturers and even between different versions of the same company's microarray; and sometimes the same identifier is reused and represents a completely different oligo, resulting in ambiguity and potentially mis-identification of the genes hybridizing to that probe. For example, the same probe was named as "GI_21070949-S" in the Illumina MouseRef-8_V1 chip but as "scl022190.1_154-S" in the later Illumina Mouse-6_V1 chip. The lack of permanency of internal identifiers causes difficulties when combining clinical microarray data collected over time using different versions of the chips. Moreover, internally created identifiers can sometime fail to satisfy the one-to-one mapping rule. For instance, the same probe was identified degeneratively as both "GI_28476905" and "scl0076846.1_142" on the Illumina MouseRef-8_V1 chip. Thus, without a central authority to approve the uniqueness, internally created identifiers can fail to be globally unique, and sometimes even locally unique. However, such a central approval process may not be socially feasible.

**Figure 1:**
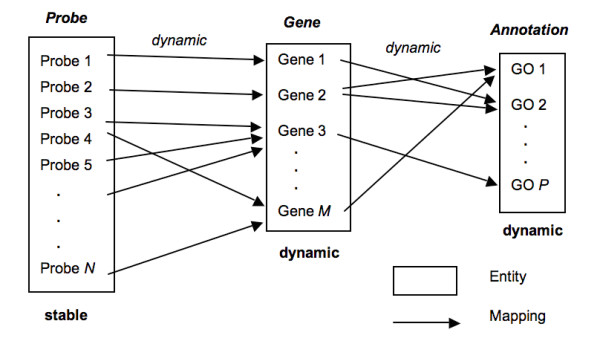
**The mapping from probes to genes and annotations**. Note that both genes (for example the definition of a gene or its representative sequence) and annotations (for example, functional annotation by Gene Ontology) are dynamic; so are the mappings among them. Only the probe sequence is stable over time.

Utilizing external gene identifiers apparently both solves the cross-manufacturer data integration problem and the permanency of the identifier. However, it blurs the distinction between oligo probes and genes. Although a good probe sequence should uniquely hybridize with only the RNA from a single gene, in practice this is very difficult to achieve across a genome due to the presence of alternative transcripts, gene families, conserved domains, and other sequences that, if included in the probe, result in cross hybridization with RNA species from different genes. Moreover, the probe-to-gene mapping is dynamic because of the continual improvement of genome annotations (Figure 1). Consequently, a probe mapped to a gene last year might map to a different gene based on the most recent evidence in the genome database. Also, multiple distinct oligos can be used as probes for the same gene transcript, again failing the uniqueness criterion. Thus, the "permanent identification" of a probe by using an external gene identifier also presents a set of limitations. Figure 1 summarizes the mapping relations from probes to genes, and from genes to annotations. Both the gene (intron and exon structure) and functional annotations to that gene may change as our knowledge of that gene structure and function improves. Only the sequence of the probe is stable over the time after the microarray is manufactured. For these reasons, major commercial vendors now release the exact sequence of each of their probes, and reporting probe-level data from microarray hybridization and analysis results in publications and public repositories is preferred [2]. The probe-level expression data requires less inference and enables researchers to reanalyze results with the latest mappings of probes to annotation resources such as RefSeq [3]. Moreover, the reporting of results at the level of the probe facilitates the aggregation of those results across microarray platforms [4,5]. Further, a universal naming convention based on the sequence of the probes will enable better probe-level studies of microarrays, especially as tiling arrays and exon arrays are becoming popular.

### Alternative approaches

A general solution to identifier-problems in biological databases is the Life Science Identifier (LSID). LSIDs are a concatenation of an identifier with its database context using the syntax of urn: lsid:<authority>:<lsid_namespace>: <identifier>:<version> to ensure the global uniqueness of the identifiers [6]. For example, the Illumina 50-mer probe "ri|E030045A12| PX00206L14| AK053222| 1725-S" on the Mouse-6_v1 chip, is represented by the LSID as a 73-character string of "urn:lsid:illumina.com:Mouse-6_v1:ri|E030045A12| PX00206L14| AK053222| 1725-S". In many cases, the resultant long string is cumbersome to use and for oligonucleotides does not allow us to easily identify degenerate names (the subset of names that are pointing to a single, identical sequence). This identifier is very useful from the standpoint of identifying the origin of the oligo (the vendor) and the version of the microarray, and from a system to system interoperability standpoint will preserve that information independent of other shared header information. However, it does not help resolve identical sequences across vendors or between versions of the chips. Instead of dealing with the general problem of identifying all biological entities, we were seeking a specific solution to a simpler problem, that of finding a common identification and representation mechanism for oligos across manufacturers.

An additional real world problem is that identifiers can be corrupted when transmitted across platforms [7]. For example, the tumor suppressor gene DEC1 (deleted in esophageal cancer 1) was being automatically converted to "1-DEC" by Microsoft Excel. Zeeberg et al. [7] offered an Excel-specific work-around – we were seeking a more general solution for corruption detection.

In summary, we have developed a permanent identifier schema for oligos, one that is deterministically unique both locally and globally. The proposed identifier should not need a central agency to approve its uniqueness, be easy to generate, and be resistant to transmission accidents. We report such a new naming schema as nucleotide universal identifier (nuID). We demonstrate the utility of the nuID with the Illumina microarray systems. The same identifier scheme can be applied to Affymetrix, Agilent and other oligo-based microarrays. The nuID schema enables the data management system to report hybridizations, annotate microarray elements, and model the gene-to-probe bindings explicitly at the probe-level, and we believe it is ideally suited for labeling the identity of microarray probes.

## Results

### Nucleotide Universal Identifier (nuID)

**Figure 2:**
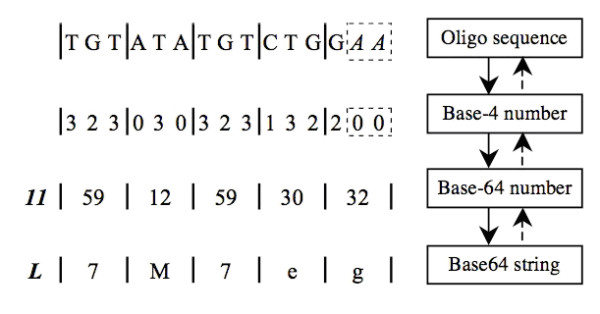
**The encoding and decoding process of nuID**. The solid arrows represent the encoding process, and the dashed arrows represent the decoding process. The bold-italic number 11 is the numeric value of the checking code "L". The "AA" at the end of sequence is the padded nucleotides.

Inspired by the fact that the sequence of monomer units is the definition of identity, we devised a simple encoding algorithm to convert the sequence itself into a universal identifier. Inversely, the sequence can be decoded unambiguously from the identifier (Figure 2). That is, the nuID compression scheme is loss-less, ensuring a one-to-one relationship between an nuID and a probe sequence.

In addition, we added an error detection code to the identifier. For applications where users want to keep the probe sequence confidential, encryptions can also be easily added to the encoding algorithm. We list a few examples of both Affymetrix and Illumina probes in Table 1.

**Table 1:** Examples of nuIDs

Array Type	Manufacturer's Proprietary Identifier	Nucleotide Sequence	nuID
Affymetrix Human	206064_s_at_probe1	TGTATATGTCTGGTTTTCTTACCCC	a7M7ev98VQ
Illumina Human	GI_23097300-A	GCTTCACTCGCTTCCCAGGGGCTCCGTTCACCAACTACATGAGCTACACG	cn0dn1Sqdb0UHE4nEY
Illumina Mouse	TRBV23_AE000664_T_cell_receptor_beta_variable_23_106-S	GACCCTTCGAAGTGAAAGAACACAGTCATGTTATATGGTATAGTCATGGT	9hX2C4CBEtO8zrMtOs

### Error detection and identification of nuID

Although the encoding and decoding of the nuID is an error-free process (i.e., 100% faithful conversion between the nuID and the oligo sequence), the nuID, same as any identifiers, can be accidentally modified during the data transmission process. Besides typos, Zeeberg et al. [7] demonstrated that applications, such as Excel, can accidentally modify the identifiers. Although such accidents are rare in reality, they have serious consequences when they occur. Another real life situation is that the users might mistakenly take another kind of identifier (a text or string) and request an nuID conversion to an oligo sequence. In order to solve these problems, we have devised a build-in mechanism to make the nuID algorithm "intelligent".

The error-checking concept is well known in other engineering fields and is nearly ubiquitous in communications; this concept has not, to our knowledge, previously been applied to genomic identifiers. We have chosen a standard checksum error-checking scheme for its simplicity and efficiency. In order to estimate the real error detection power of the current implementation (*N *= 21, see Equation 4 in Methods), we simulated two scenarios: mutating a number of characters in a legitimate nuID; and randomly generating a "spoof" nuID using 95 printable ASCII characters. Under each scenario, the simulation was repeated for 10^5 ^times, and the estimated error detection rate is shown in Table 2. The checksum algorithm can detect the majority of errors in both scenarios, and the detection power increases with the number of mutated characters. The error detection rates of the nuID (*N *= 21) are always over 97.5% in the mutation scenario. Considering that this type of mutation is rare in reality, the error detection rates of the nuID should be sufficient for most applications. For the second simulation scenario, the detection rate of a random sequence is over 99.9%. This means a legitimate nuID can almost always be differentiated from a common text or sequence. If an application has a more stringent error-checking requirement, one more digit of checking code can be added. We have named this identifier nuID2 (*N *= 1344) to distinguish it from our standard encoding scheme. A more sophisticated error detection algorithm can also be applied, and the detection power can be further improved. However, these techniques will add to the length of the identifier and increase the computation required to encrypt and decrypt the probe sequences.

**Table 2:** The error detection power of the nuID checksum algorithm (*N *= 21)

*L*	1-character	2-character	3-character	Random
25mer	0.97780	0.97918	0.98689	0.99924
50mer	0.97724	0.97838	0.98607	0.99997
100mer	0.97894	0.97825	0.98617	1*

*L *and *N *are defined in Equation (3) and (4) in Methods. The column "1-character" is the error detection rate of an nuID with only one character mutated. Similar definition for column "2-character" and "3-character". "Random" column is error detection rate of a random ASCII string. The optimum detection power is 1.0.

* We realize the detection of nuIDs for 100mers is not guaranteed, but in none of our simulations did we ever encounter a randomly assembled string that was a valid nuID.

### Encryption capability of nuID

For some applications, users may not want the probe sequence public. In this case, an additional encryption step can be added to the nuID. Suppose *f*(*x*) and *f*^-1^(*x*) is the encryption and decryption algorithm respectively. Then following Equation (1) and (2), we can easily encrypt and decrypt the nuID.

x'nuID=f(xnuID)
 MathType@MTEF@5@5@+=feaafiart1ev1aaatCvAUfKttLearuWrP9MDH5MBPbIqV92AaeXatLxBI9gBaebbnrfifHhDYfgasaacH8akY=wiFfYdH8Gipec8Eeeu0xXdbba9frFj0=OqFfea0dXdd9vqai=hGuQ8kuc9pgc9s8qqaq=dirpe0xb9q8qiLsFr0=vr0=vr0dc8meaabaqaciaacaGaaeqabaqabeGadaaakeaacqWG4baEcqGGNaWjdaWgaaWcbaGaemOBa4MaemyDauNaemysaKKaemiraqeabeaakiabg2da9iabdAgaMjabcIcaOiabdIha4naaBaaaleaacqWGUbGBcqWG1bqDcqWGjbqscqWGebaraeqaaOGaeiykaKcaaa@3EF5@

xnuID=f−1(x'nuID)
 MathType@MTEF@5@5@+=feaafiart1ev1aaatCvAUfKttLearuWrP9MDH5MBPbIqV92AaeXatLxBI9gBaebbnrfifHhDYfgasaacH8akY=wiFfYdH8Gipec8Eeeu0xXdbba9frFj0=OqFfea0dXdd9vqai=hGuQ8kuc9pgc9s8qqaq=dirpe0xb9q8qiLsFr0=vr0=vr0dc8meaabaqaciaacaGaaeqabaqabeGadaaakeaacqWG4baEdaWgaaWcbaGaemOBa4MaemyDauNaemysaKKaemiraqeabeaakiabg2da9iabdAgaMnaaCaaaleqabaGaeyOeI0IaeGymaedaaOGaeiikaGIaemiEaGNaei4jaCYaaSbaaSqaaiabd6gaUjabdwha1jabdMeajjabdseaebqabaGccqGGPaqkaaa@4109@

where *x*_*nuID *_is the base-64 number corresponding to the nuID, and x'nuID
 MathType@MTEF@5@5@+=feaafiart1ev1aaatCvAUfKttLearuWrP9MDH5MBPbIqV92AaeXatLxBI9gBaebbnrfifHhDYfgasaacH8akY=wiFfYdH8Gipec8Eeeu0xXdbba9frFj0=OqFfea0dXdd9vqai=hGuQ8kuc9pgc9s8qqaq=dirpe0xb9q8qiLsFr0=vr0=vr0dc8meaabaqaciaacaGaaeqabaqabeGadaaakeaacqWG4baEcqGGNaWjdaWgaaWcbaGaemOBa4MaemyDauNaemysaKKaemiraqeabeaaaaa@342B@ is the encrypted base-64 number, which can be further transformed as a Base64 encoding identifier, i.e. the encrypted nuID. Different encryption algorithms can be adopted for different application requirements. We have not released code with encryption algorithm and are providing it as a convenience for applications where it is necessary to obfuscate the identity of the probe.

### Application of nuID to Illumina microarrays

Constructing and managing identifiers on microarrays is a challenging problem. The design of the Illumina Mouse microarray version 1, for example, agglomerated thirteen sequence database sources by taking identifiers from each of those sources and appending additional tags. Illumina expression microarrays have employed two schemes for identifying probes: "Probe Id" and "Target Id". The "Probe Id" is a probe-level identifier. It is a number proprietarily assigned by Illumina Incorporated for the internal decoding process of beads and may potentially change between different versions and occasionally between different batches of microarrays; thus, "Probe Id" is not recommended for external reporting (personal communication with Illumina Technical Support). The "Target Id" is a gene-level identifier, either a Genbank GI number (Mouse version 1), or a manufacturer assigned internal identifier (version 2 or later). Note that one "Target Id" can correspond to several "Probe Ids" based on the best probe-to-gene mapping. Neither "Probe Id" nor "Target Id" can satisfy the properties of the ideal nomenclature system, as was discussed in the introduction section.

By applying the nuID system to Illumina microarrays, we are able to achieve the following four aims.

(1) There is a one-to-one mapping between the identifier and probe sequence. We are able to uniquely identify and thus quantify different probes of the same gene, which enables us to search for evidences of alternative splicing. Moreover, the nuID makes the external reporting of microarray results feasible at the probe level.

(2) The nuID can be directly converted to the probe sequence, and be used to get the most updated RefSeq matches and annotations (see the paper companion website). The available sequence also enables future studies to model the potential cross-hybridization or binding affinity of the probes explicitly.

(3) The nuID identifiers are permanent and consistent. The same probe in different versions of the microarrays always has the same identifier.

(4) A shared annotation database of 50-mers, which is independent of array versioning and manufacturers, was created. The use of nuIDs will simplify and standardize the maintenance and usage of probe annotations.

## Discussion and conclusion

Similar to the manufacturer independent chemical formula (for example, H_2_O) used to identify a chemical compound, we formulated a universal identifier that guarantees global uniqueness for oligonucleotides. Mathematically, the nuID encoding of the sequence is lossless and there is a one-to-one correspondence of the probe sequence to an nuID. A significant advantage of nuIDs is that a sequence can be converted to an nuID by any individual yet is guaranteed to be globally unique and interoperable; no central authority is necessary to assess the uniqueness and approve the legitimacy of the identifier.

The nuID schema has three significant advantages over using the oligo sequence directly as an identifier: first it is more compact due to the base-64 encoding; second, it has a built-in error detection and self-identification; and third, it can be encrypted in cases where the sequences are preferred not to be disclosed.

By incorporating the nuID naming scheme into the probe annotation workflow, it is easier to build a generic annotation pipeline that is independent of manufacturer and makes the maintenance of annotations independent of the manufacturer. More importantly, we are able to make the probe-to-gene mapping explicit. Given the proliferation of gigabit/second interconnects and the efficiency of modern computer architectures, the overhead cost of using nuIDs as the primary identifier for a sequence versus an 8 or 10 digit number is trivial. The encoding and decoding algorithm of the nuID has been implemented in R, Perl, and ColdFusion and can be downloaded from the companion website listed in this article.

We have demonstrated the utility of this encoding schema with Illumina microarrays. As shown in Table 1, it is actually more compact and efficient for the 25-mer based Affymetrix system. The use of nuIDs will eliminate the need to maintain the probe-to-sequence lookup table currently in the Bioconductor distribution. The nuID and consequently the availability of the probe-sequence itself, enables one to explicitly model individual probe behaviors, which can be used in probe-selection [8], alternative-transcription modeling [9], background binding affinity modeling [10], or probe-affinity modeling [11]. In addition to microarray probes, we have found that the nuID, without modification, can also be used to name SAGE [12] tags. For example, the 17-nucleotide SAGE tag of "GCTGATATTTAAAAGAG" can be identified by the nuID of "BnjPwCI", with the same advantages as discussed in the paper. We also expect that nuIDs will be used to cross-reference databases, including commercial catalogs and inventory control systems.

The nuID scheme is simple to implement, but was not obvious to conceive. The general concept and implementation of the nuID is also applicable to oligopeptides and other objects that are a sequence of defined monomers. Another further application, that has not escaped our notice, is that the encoded string allows us to quickly and easily identify whether the oligos are identical or frameshifted from each other using standard bitstring comparison routines.

## Methods

Base64 encoding is a scheme that encodes binary data as a string composed of a set of 64 characters. It is widely used in Internet data transfer for its efficiency and ease of use. The transformation of DNA sequence into Base64 results in a three-fold, lossless compression of the nucleotide sequence.

To encode a DNA oligonucleotide sequence, we use a variation on standard Base64 encoding, where "+" and "/" are replaced with "_" and "." to avoid misinterpretation by some analytical applications, particularly with the "+". Similar to the Base64 standard, the character set A-Z, a-z, 0–9, "_" and ".", represent the base-64 numbers of 0–63. The following steps are used to convert a nucleotide sequence to Base64. First, four nucleotides **A**, **C**, **G **and **T **(**U**) are mapped to the base-4 numbers 0, 1, 2 and 3; then three base-4 digits are combined as one base-64 digit; finally, the base-64 number is mapped to the Base64-encoded character set. This is the essence of the nuID coding scheme. Note that since "**A**" is mapped to 0, sequences that are not divisible by three will appear to have been padded with "**A**"s. In order to make the identifier non-degenerate, we therefore need to add a 'length' bit, to specify whether in the original sequence the last triplet had 1,2 or 3 real nucleotides. Also, the basic encoding steps do not incorporate self-identification or error checking, which are also desirable attributes for a coding scheme.

By incorporating error checking directly into the nuID, errors occurring during the transmission of the nuID can be immediately detected. This also allows us to differentiate an nuID from an arbitrary character string, providing a means for the auto-detection of nuIDs. Furthermore, because nucleotide "**A**" is padded at the end of nucleotide sequence to make the sequence length divisible by three, we need to record the number of padded "**A**"s. To fulfill these three requirements, we added a checking code *C *at the beginning of the Base-64 encoded string. The base-64 numeric value of the checking code *C *is:

*c *= 3 * *n *+ *p*, *p *∈ {0, 1, 2}

n=∑i=1Ldi(mod⁡N)
 MathType@MTEF@5@5@+=feaafiart1ev1aaatCvAUfKttLearuWrP9MDH5MBPbIqV92AaeXatLxBI9gBaebbnrfifHhDYfgasaacH8akY=wiFfYdH8Gipec8Eeeu0xXdbba9frFj0=OqFfea0dXdd9vqai=hGuQ8kuc9pgc9s8qqaq=dirpe0xb9q8qiLsFr0=vr0=vr0dc8meaabaqaciaacaGaaeqabaqabeGadaaakeaafaqabeqacaaabaGaemOBa4Maeyypa0ZaaabmaeaacqWGKbazdaWgaaWcbaGaemyAaKgabeaaaeaacqWGPbqAcqGH9aqpcqaIXaqmaeaacqWGmbata0GaeyyeIuoaaOqaaiabcIcaOiGbc2gaTjabc+gaVjabcsgaKjabd6eaojabcMcaPaaaaaa@3F60@

where *c *is the base-64 numeric value of checking code *C*, *p *is the number of padded "**A**"s, *n *is the checksum residue of module *N*, and *L *is the length of base-64 number *d*_i_. To maximize the power of error detection, we selected *N *= 21, and thus *c *∈ {0,...,62}. Figure 2 shows an example of encoding and decoding process of oligonucleotide sequence "TGTATATGTCTGG" and its corresponding nuID "L7M7eg". In this example, the oligonucleotide sequence is first padded with two (*p *= 2) "**A**"s to make the sequence length divisible by three; then every three nucleotides are transformed to a base-64 number; based on Equation (4), the checksum of adding all 5 Base64 numbers is 192, taking the modulo 21 and getting *n *= 3; finally, based on Equation (3), multiplying *n *by 3, and adding the number of padding bases *p *(*p *= 2) to get the checking code value *c *(*c *= 11).

The decoding process is just the reverse of encoding. The checking code is separated first and converted to a numeric value *c*. Based on Equation (3), we can calculate the checksum residue, *n*, and the number of padded "**A**"s, *p*. In parallel, the checksum residue, *n*, can also be calculated based on Equation (4) by using the numeric values of the encoded base-64 string. If the two estimated *n *based on Equation (3) and (4) are different, an error will be reported. It indicates the input string is not an nuID or that there is an error in the storage or transmission of the identifier. The algorithms above are implemented in R and Perl programming language and also as a webservice for broader accessibility (see the paper companion website).

## Availability

The encoding and decoding algorithm is implemented in R, Perl, and ColdFusion. Supplemental data is at [13] and also as a web server and a webservice accessible at [14].

## Competing interests statement

The authors declare that they have no competing interests. Findings from this paper have been reported to the Technology Transfer Program of Northwestern University.

## Authors' contributions

PD and SML got the ideas of the nuID through discussion. PD implemented the nuID encoding and decoding algorithms in R (in the BioConductor *lumi *package) and produced nuID indexed Illumina annotation packages. WAK implemented the nuID encoding and decoding algorithms in Perl and ColdFusion, and set up webservices for broader accessibility. SML drafted the manuscript. All the authors participated in the manuscript preparation and modification. All the authors read and approved the final manuscript.

## Reviewer's comments

### Review 1

Dr. Itai Yanai, Department of Molecular and Cellular Biology, Harvard University

In this manuscript Lin and colleagues address a problem in microarray probe nomenclature. As microarray design is becoming more and more commonplace, new probes are being designed at an increasing rate. Likewise, the annotation of the RNA species targeted by the probes is quickly being updated. In such a situation, the authors point out, the only stable entity

is the sequence of the probe. They propose that the probe ID should be based upon the actual sequence, encoded in base-64 along with a checksum to detect corruption of the identifier.

I agree with the author's assessment of the situation in probe nomenclature. Furthermore, I plead guilty myself in introducing some probe identifiers that perhaps may have been better named. However, many probe identifiers have the usefulness of immediately communicating the target they were from the encoded-sequence identifier. Nevertheless, the proposed system is potentially useful in the modern era of integrating microarray data based upon the probe sequence alone.

Perhaps one compromise would be for each lab to design two probe identifiers per prone – one formal identifier in the proposed format to be used during publication, and a second identifier for internal use in the lab based upon current annotations. The second informal identifier can change with time while the first, of course, is fixed and true.

#### Author's response

*We completely agree with the reviewer's opinion. In addition to the deterministically unique and permanent identifier of nuID for computers, a more flexible and less stringent label can be used to facilitate human communications*.

### Review 2 (nominated by Dr. Mark Gerstein)

Dr. Rong Chen, Department of Medical Informatics, Stanford University

This paper addresses a very important problem, standardizing the probe naming in microarrays. It describes an efficient encoding algorithm to transfer a probe sequence into a universal identifier with automatic error detection. It will greatly facilitate microarray data re-analysis and cross-platform comparison. I have no reservation in recommending it for publication.

Here are some suggestions to make it more readable and helpful to the community. The equation 4 in the method section is not very understandable. Although the authors have provided a R function, a web server for batch encoding and decoding would be more convenient.

#### Author's response

*We followed the reviewer's suggestion and rewrite the description of Equation 4 by adding an example to explain it. We also added a web service for batch conversion of nuIDs*.

### Review 3 (nominated by Dr. David Lipman)

Dr. Gregory Schuler, NCBI

This reviewer provided no comments for publication.
